# Vogt-Koyanagi-Harada disease: a retrospective and multicentric study of 41 patients

**DOI:** 10.1186/s12886-020-01656-x

**Published:** 2020-10-07

**Authors:** K. Diallo, S. Revuz, G. Clavel-Refregiers, T. Sené, C. Titah, M. Gerfaud-Valentin, P. Seve, R. Jaussaud

**Affiliations:** 1 Department of Internal Medicine, Nancy University Hospital, Nancy, France; 2 Department of Internal Medicine, Metz Private Hospital, Metz, France; 3 Department of Internal Medicine, Rothschild Hospital Foundation, Paris, France; 4 Department of Ophthalmology, Rothschild Hospital Foundation, Paris, France; 5 Department of Internal Medicine, Lyon University Hospital, Lyon, France

**Keywords:** Vogt-Koyanagi-Harada, Uveitis, Poliosis, North African

## Abstract

**Background:**

East and South East Asian subjects as well as Amerindians and Hispanic subjects are predominantly affected by Vogt-Koyanagi-Harada disease.

In Europe, only few studies have described the clinical features and treatment of this disease, especially in France.

**Methods:**

This retrospective case series was based on data collected from patients with a VKH disease diagnosed from January 2000 to March 2017, provided by three French Tertiary Centers.

**Results:**

Forty-one patients (16 men and 25 women) were diagnosed: average age at diagnosis was 38.7 years. Patients were mainly from Maghreb (58%), but ethnic origins were multiple. Pleiocytosis was observed in 19 cases (63%) and 17 out of 41 patients showed audio vestibular signs (41%), and 11 showed skin signs (27%). Thirty-four were treated with corticosteroids (83%), 11 with an immunosuppressant treatment (27%) and 5 with biological therapy drugs (13%). Relapse was observed in 41% patients, even though final average visual acuity had improved. We did not find any significant clinical difference in the population from Maghreb compared to other populations, but for age and sex trends, since there was a majority of younger women.

**Conclusion:**

We report here the second largest French cohort reported to date to our knowledge. The multiethnicity in our study suggests that VKH disease should be evoked whatever patients’ ethnicity.

## Background

The Vogt-Koyanagi-Harada (VKH) disease is a bilateral granulomatous panuveitis with potential systemic involvements: neurological disorders (cerebrospinal fluid analysis shows pleiocytosis in about 80% of cases), otological disorders (hearing loss, dizziness (70%) and tinnitus (42%)) and dermatological disorders such as vitiligo, poliosis and alopecia (10 to 63%) [1]. The disease is mediated by Th1 lymphocytes targeting melanocytes [2]. The origin of this affection remains unknown, though many infectious triggers have been hypothesized [2–4]. The association with HLA DR4/ HLA DRB1–04*05 has been reported in the Japanese population [5]. VKH disease mainly affects subjects from East and South East Asia, as well as Amerindian and Hispanic patients [1, 6]. Median age at diagnosis is around 40. Only few studies are available to estimate prevalence of ethnic characteristics in the European population [7–10]. The aim of this study was to describe patients’ epidemiological characteristics in clinical French referral centers.

## Methods

### Study design

This retrospective, multicentric-case series was based on data collected from patients with a VKH disease diagnosed from January 2000 to March 2017 provided by three French tertiary Centers (Lyon University Hospital, Rothschild Hospital Foundation in Paris, Nancy University Hospital). Data was collected in each center. At the Nancy University Hospital cases carrying the diagnosis code “other chorioretinitis and other iridocyclitis” were collected from the Department of Medical Information in France and all cases of VKH disease were selected. At the Lyon University Hospital and at the Rothschild Hospital Foundation in Paris cases were selected by doctors managing patients with a suspect diagnosis. The selected cases met the revised diagnostic criteria for VKH disease: report of an international nomenclature committee [11]. Four patients were excluded because of missing data. As the data was retrospectively collected the Jarde law was not appliable and no consent was requested.

### Patient selection

Among patients with a possible VKH disease diagnosis was reported by the Medical Data Department and physicians; those with a definite diagnosis based on the revised diagnostic criteria for Vogt-Koyanagi-Harada disease, report of an international committee on nomenclature, were included [11]. As a matter of fact, ophthalmic criteria are clinical and paraclinical: a bilateral ocular involvement depending on the stage of disease. Indeed, early manifestations of the disease are choroiditis (with or without anterior granulomatous uveitis, vitreous inflammatory reaction, or optic disc hyperhemia), which may be manifest as focal areas of subretinal fluid or bullous polycyclic exudative retinal detachments. Paraclinical investigations can contribute to make the diagnosis:

- fluorescein angiography showing focal delayed choroidal perfusion, multiple areas of pinpoint leakage, large placoid areas of hyperfluorescence, pooling within subretinal fluid, and optic nerve taining,
- ICGA (indocyanin green angiography) is generally employed in the study of choroidal vasculature choroidal stroma and evolutionary monitoring of the disease [12],
- Enhanced-depth imaging spectral-domain OCT improves the visualization of the choroid and its thickness [13, 14].

Late manifestations of the disease are signs of ocular depigmentation: either sunset glow fundus, Sugiura sign or other ocular signs including nummular chorioretinal depigmented scars, retinal pigment epithelium clumping and/or migration, or recurrent or chronic anterior uveitis.

Contrarily to the chronic form of the disease, patients are diagnosed at the early stage of disease or at acute early onset of the disease when diagnosis and treatment are performed within 3 weeks of symptoms [15].

The presentation was considered as “complete” when the patient presented an ocular involvement with both neurological or otologic and dermatological involvement; as “incomplete” if the patient presented with an ocular involvement and another involvement whether otologic, neurological or dermatological and as “possible” if the ocular presentation only was present. Differential diagnoses, especially infectious and inflammatory ones were excluded by no harmonized investigations, mainly: Lyme’s disease, syphilis and HIV serologies, search for tuberculosis (Mantoux tuberculin skin test or interferon-gamma release assays), and search for sarcoidosis (angiotensin converting enzyme and chest computed tomography).

A pleiocytosis was defined by > 10 cells/mm3 at the cerebrospinal fluid analysis.

A treatment failure was defined as the presence of an ophthalmic inflammatory manifestation in at least one eye (recurrence or chronic inflammation) with corticosteroid therapy tapering or withdrawal leading to therapeutic intensification (increase of corticosteroid therapy or addition of an immunosuppressive agent) [16].

### Data collection

Data was collected from December 2016 to April 2017 on an internet-based survey on WebQuest or sent by internet available in Supplementary File. Patients showing a VKH disease from January 2000 to March 2017 were included.

### Statistical analysis

Quantitative variables are presented as mean +/− standard deviation or as median and range and were compared using analysis of variance. Qualitative variables are presented as number and percentage and were compared using a Chi2 test (or Fischer’s exact test if the criteria for a Chi2 test were not fulfilled). A value of *p* < 0.05 was considered as significant. Statistical analysis was realized with R 3.3.1 statistical software (http://www.r-project.org).

## Results

Forty-one patients (25 women and 16 men) were included. The main characteristics are reported in Table 1.

**Table 1:** Main epidemiological characteristics

Characteristics	Patients n/41 (%) – Means	p
Gender		
- women	25/41 (61)	0.15
Ethnic origin:		
- Japanese	1/41 (3)	0.27
- South East Asian (except Japan)	7/41 (17)	
- North African (Maghreb)	24/41 (58)	
- Caucasian	8/41 (19)	
- Hispanic	1/41 (3)	
Age at diagnosis	38.7 [10–74]	
HLA DRB1–04*05/ HLA DR4		
- Positive HLA DRB1–04*05	4/10 (40)	0.5
- Positive HLA DR4	1/9 (11)	0.01

Mean age at diagnosis was 38.7 years (10–74) and the median of follow-up was 29.7 months (1–140). North African was the main represented ethnicity 58% (24/41) followed by South East Asian 49% (20/41), Caucasian 20% (8/41) and Hispanic 2% (1/41). Thirty patients were diagnosed at the acute early onset stage of the disease (73%). Nine patients had a complete presentation (22%), 19 an incomplete presentation (46%) and 13 had a possible syndrome (32%). All patients with a dermatological manifestation were diagnosed at the late stage of the disease. First entry in the medical care system was mainly by the Ophthalmology department (92%, 37/41) and patients were mostly followed by both Ophthalmology and Internal Medicine departments (61%, 25/41).

### Ophthalmic manifestations

**Fig. 1:**
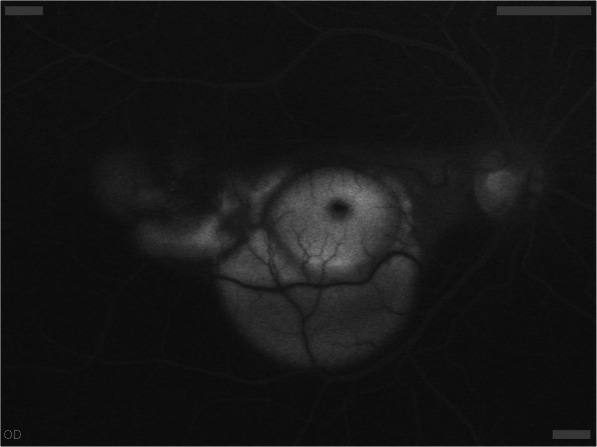
Exudative detachment of the neurosensory retina seen on fluorescein angiography, right eye

**Fig. 2:**
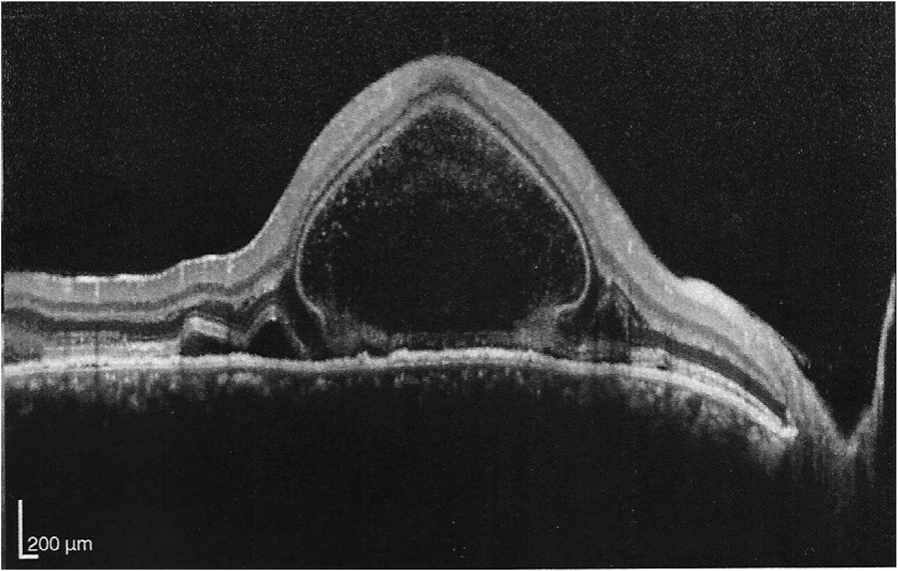
Exudative detachment of the neurosensory retina seen on the optical coherence tomography, right eye

**Fig. 3:**
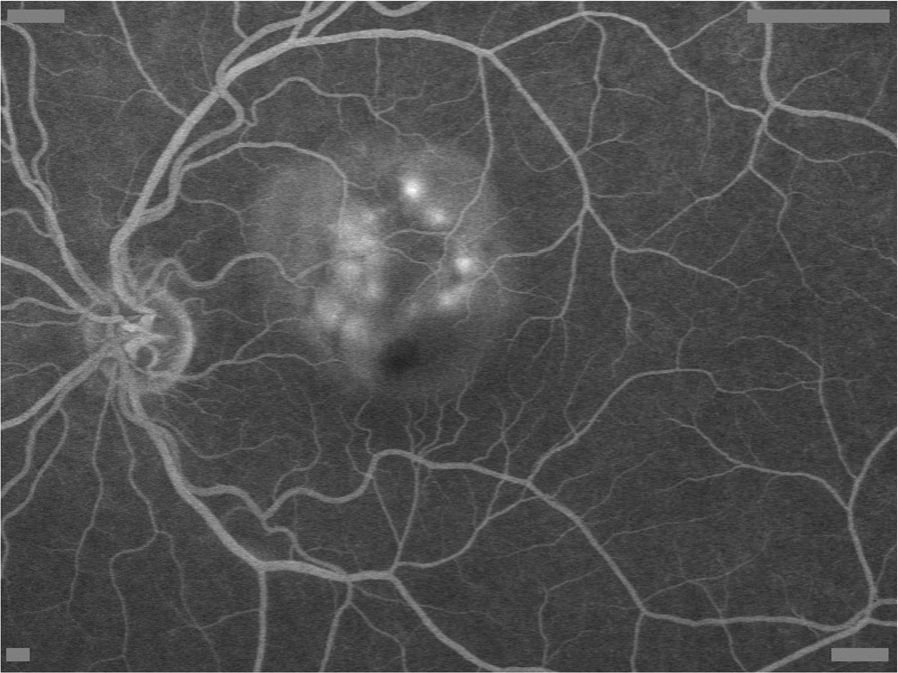
Hyperfluorescent leaking dots « pinpoints » on the fluorescein angiography, left eye

Main ophthalmic manifestations were: exudative detachment of the neurosensory retina (75%, 31/41) at the fundus examination (Fig. 1) or at the optical coherence tomography (Fig. 2), hyperfluorescent leaking dots ≪ pinpoints ≫ on the fluorescein angiography (44%, 18/41) (Fig. 3) and peripapillary atrophy and depigmented small atrophic lesions at the level of retinal pigment epithelium or ≪ Sunset glow fundus ≫ (41%, 17/41). The main results of ophthalmic clinical examination and investigations are described in Table 2.

**Table 2:** Patient’s presentation

Investigations	Patients n/N (%) – Means +/− standard deviation	Patients diagnosed at the early stage n/N (%) - Means +/− standard deviation	Patients diagnosed at the chronic stage n/N (%) – Means +/− standard deviation	p
First manifestation:				
- bilateral loss of visual acuity	27/41 (66)	18/31 (58)	9/10 (90)	0.06
- unilateral loss of visual acuity	10/41 (24)	9/31 (29)	1/10 (10)	0.01
- ocular pain	1/41 (2)	0/31 (−)	1/10 (10)	
- neurological and auditory involvement	2/41 (5)	2/31 (6)	0/10 (0)	
Other	1/41 (2)	1/41 (2)	0/10 (0)	
Signs at ophthalmic clinical examination:				
- Bilateral exudative detachment of the neurosensory retina	29/41 (70)	23/31 (74)	6/10 (60)	0.05
- Accumulation of liquid in the sub retinal space	15/41 (37)	11/31 (35)	4/10 (40)	0.22
- « Sunset glow fundus »	2/41 (5)	2/31 (6)	0/10 (−)	
Initial visual acuity (/10):				
- Right eye	4.6 +/− 3.6	4.3 +/− 3.7	4.3 +/− 3.5	0.84
- Left eye	4.9 +/− 3.4	4.6 +/− 3.4	5.0 +/− 3.7	0.88
Angiography				
- Delay in choroidal perfusion, causing hypofluorescence of circumscribed areas poorly perfused	15/31 (48)	11/25 (44)	4/6 (66)	0.47
- Hyperfluorescent leaking dots (pinpoints)	17/31 (55)	16/25 (64)	1/6 (17)	0.13
- Retinal pigment epithelium clumping and/or migration	15/31 (48)	13/25 (52)	2/6 (33)	0.55
Optical coherence findings:				
- Diffuse choroidal thickening	17/31 (55)	15/25 (60)	2/6 (33)	0.36
Neurological and auditory investigations:				
- Cerebrospinal Fluid pleiocytosis	19/30 (63)	12/21 (57)	7/9 (78)	0.21
- Audiological testing with a loss of hearing	8/22 (36)	5/18 (28)	3/4 (75)	0.13

The average of initial visual acuity was 4.6/10 for the right eye and 4.9/10 for the left one.

### Extra-ophthalmic manifestations

Two patients first complained of a neurological and auditory involvement. Nine patients had a meningismus (22%). The analysis of cerebrospinal fluid was performed on 30/41 patients: among them 19 showed a lymphocytic pleiocytosis (63%). A cerebral imaging was performed on 71% of the patients (27/38) including 26 cerebral Magnetic Resonance Imaging, which did not find any specific abnormality. Seventeen patients complained of auditory and vestibular manifestations (41%): hearing loss (24%, 12/41) and tinnitus (22%, 9/41). The audiogram was performed on 22 patients and showed a hearing loss in 8 of them (36%). Eleven patients had dermatological manifestations (27%) including alopecia (5/11), vitiligo (4/11) and poliosis (6/11).

### HLA association

Among ten tested patients four were positive to HLA DRB1–04*05; one was positive to HLA DR4 out of nine tested.

### Treatments and prognosis

All but four patients were treated with high doses of corticosteroid therapy, 1 mg/kg/d prednisone or equivalent by oral route with progressive tapering (Table 3).

**Table 3:** Treatments and prognosis

Characteristics	All patients n/N (%) - Means	Patients diagnosed at the early stage n/N (%) - Means	Patients diagnosed at the chronic stage n/N (%) - Means	p
Treatment with corticotherapy:				
- Bolus	34/41 (83)	26/31 (84)	5/10 (50)	< 0.01
- Mean posology of bolus (mg/day)	730	650	550	< 0.01
- Oral corticotherapy at 1 mg/kg	37/41 (90)	26/31 (84)	8/10 (80)	
Associated treatments (first line or relapse):				
- Immunosuppressive agents:	12/41 (29)	5/31 (16)	6/10 (60)	< 0.01
Azathioprine	7/41 (17)			
Ciclosporine	1/41 (2)			
Mycophenolate mofetil	3/41 (7)			
- Biological therapies (infliximab and adalimumab)	5/42 (12)	3/31 (10)	2/10 (20)	< 0.01
Clinical relapse	18/41 (44)	11/31 (35)	7/10 (70)	0.15
Mean duration of follow-up after treatment (months)	13.3 [1–140]	30.9	44.1	
Mean posology of corticotherapy at relapse (mg)	15	18.6	4.6	
Mean duration of treatment (months)	23.4	20	36	< 0.01
Final visual acuity (/10):				
- Right eye	8.6	8.8	7.7	0.7
- Left eye	8.8	8.6	8.7	0.9
Complications:				
- Cataract	7/41 (17)	4/31 (13)	3/10 (30)	< 0.01
- Glaucoma	2/41 (5)	1/31 (3)	1/10 (10)	< 0.01

A parenteral route was first used for 66% of patients (27/41). Only one patient was treated with local corticotherapy; 11 patients (27%) received an associated immunosuppressive treatment; 6 patients started with corticosteroids associated to immunosuppressive treatment at the very beginning of treatment, and 5 patients were treated with immunosuppressive treatment after a first line of corticosteroids alone: Azathioprine (*n* = 7), Ciclosporine (*n* = 1) and Mycophenolate mofetil (*n* = 3). Five patients were treated with biological therapies (infliximab and adalimumab) (12%) as a third therapeutic line for 3 of them. Patients without recurrence were treated during less than a year. Relapse occurred in 44% of patients (18/41) at mean corticosteroid dose of 15 mg/day [1–80] and at mean 13 months after the beginning of treatment [1–48]. The 23 patients who did not relapse were followed on an average 21 month-follow-up period and the 18 patients who relapsed were followed on an average of 40.9 months (*p* < 0.01). Complications occurred (Table 3).

### Population from Maghreb

As for the main symptom (Table 4) (*p* = 0.8) and the HLA type (*p* = 1) there was not any significant difference between patients from Maghreb and other patients.

**Table 4:** Distinction of population from Maghreb

	Population from Maghreb n/N (%) - Means	Other populations n/N (%) - Means	p
Main symptom:			
bilateral loss of visual acuity	15/24 (62%)	12/17 (71%)	0.8
HLA type:			
- Positive HLA DRB1–04*05	2/24 (8%)	2/17 (12%)	1
- Positive HLA DR4	0	1/17 (6%)	
Gender:			
women	18/24 (75%)	7/17 (41%)	0.06
Age at diagnosis	34	44	0.06

In the population from Maghreb, we found trends toward more women (18/24 versus 7/17 (*p* = 0.06)) and younger age (34 versus 44 years at diagnosis (*p =* 0.06)) as compared to other populations with borderline statistical significances.

## Discussion

VKH disease is a rare disease and most of epidemiological data has been described for Asiatic, Hispanic, and Amerindian populations but not so for patients from Northern Africa. To our knowledge, our study is the second largest French cohort [8, 10, 17]. The low prevalence of the disease in Europe explains the low number of patients by center [18].

Apart from ethnicity, epidemiological data is the same as that of other populations’ [2, 6] with 60% of women and a mean age 38.7 at diagnosis. In our study the prevalence of the association with HLA DR4/ HLA DRB1–04*05 agrees with the literature data, the presence of this allele being associated with a higher risk of developing this disease.

As described by Lavezzo et al. and Ohno et al., the meningeal involvement is variable (63% of cases) like the clinical meningismus [1, 19]. In our study cerebral imaging did not find any significant sign, since its role is not defined in VKH disease. Although this investigation is normal in most cases, it seems relevant to exclude differential diagnoses. The otologic involvement in our population is the same as that of non-Hispanic populations’ [6]. This involvement is typical (75% of cases with a mean hearing loss of 30 dB) but seldom symptomatic [6]. While VKH disease could usually be confidently diagnosed by uveitis specialists based on ocular presentations, a search of systematical involvement, in particular an audiogram and/or a lumbar puncture, might be helpful for physicians who are less familiar with this disease. The prevalence of cutaneous involvement is closed to the non-Japanese populations’ [20]. Skin involvement prevails in pigmented populations from 10 to 63% [6]. Poliosis, vitiligo and alopecia are late manifestations of the disease. Most of our patients were diagnosed at an early stage, but a specific attention should be given to the extra ophthalmic manifestations at diagnosis of first ophthalmological manifestation and during follow-up to avoid a late-stage diagnosis. In our study, we did not notice any delay between the first manifestation of the disease and the ophthalmic involvement that led to the ophthalmic examination, but most of our patients presented the ophthalmic signs first (93%, 38/41).

Sasamoto et al. had a better outcome with high doses of systemic corticosteroid therapy [21]. Oral prednisone equivalent is usually prescribed initially at a dosage of 1 to 1.5 mg/kg/day with progressive tapering [17, 22]. In serious situations, methylprednisolone can be used at 1 g/day for 3 to 5 days [2]. The corticosteroid therapy duration is not codified, but in our study a few patients showed persistent inflammation signs at 6 months [21]. Treatment should be adapted to each patient depending on the clinical outcome. An early cessation is associated with an elevated rate of treatment failure [23]. Surprisingly, in our study treatment failures occurred from 1 to 48 months after the beginning of treatment, with a mean of 13 months, at a high level of corticosteroid of 15 mg a day, though the literature suggests a decrease of corticosteroid therapy with a minimal duration of 6 months [24]. Nevertheless, we believe that prognosis should have been different if immunosuppressive therapy had been initiated at first line [25, 26].

The sooner the diagnosis, the better the disease outcome (visual acuity ≥5/10 after treatment) [1]. This data was found in our cohort with a positive average profit by eye after treatment. An immunosuppressive treatment was introduced in 27% of cases as first line of treatment or in case of failure. Ciclosporine, Azathioprine, Cyclophosphamide, Methotrexate and Mycophenolate mofetil are active therapeutics in this disease [2, 27–29]. A recent Mexican study did not find benefit to adding early immunosuppressive treatment in terms of final visual acuity or of development of visually significant complications [25]. In contrast, other studies found a preeminence of immunosuppressive agents (Mycophenolate mofetil, Ciclosporine A, Methotrexate, and Azathioprine) over corticosteroids alone, while others were more reserved [26, 30]. Finally, biological therapies are used in non-infectious uveitis including VKH disease [31] but the treatment length in VKH disease is still debated.

Patients diagnosed at early stage presented mainly unilateral loss of visual acuity (*p* = 0.01). They were treated more often with bolus of corticosteroid (*p* < 0.01) and second line agents (*p <* 0.01). Compared to patients who were diagnosed at the early stage of disease, patients diagnosed at the chronic stage of the disease were treated longer: 36 months instead of 20 (*p <* 0.01). Complications were also more frequent in this group.

As in other French series, our study included most patients from Maghreb among a multiethnic population [8, 10]. Our prognosis seems better than in North African studies, which are more homogeneous as for ethical origin. Alaoui et al. reported about 8 women: 5 were cured without any relapse and only two were still treated with corticosteroids. Khairallah et al. found a good outcome for 59% of patients maintaining a visual acuity of 20/40 or better [32]. Boutimzine et al. observed 87.5% of patients with a visual acuity of 5/10 or better after a mean follow-up of 6 years [33]. We failed to find any significant clinical difference in the Maghreb population, but for age and gender trends. However, we report here one of the largest case reports about Maghreb to our knowledge [32–34] suggesting a higher prevalence in this population. Indeed, initial clinical presentation seems more severe in this population, especially the ophthalmic presentation, even if we did not find any clinical significance [35]. In our study the initial visual acuity was low, and this data was associated with a worse prognosis factor [36]. Surprisingly, as opposed to Read et al. who observed 51% of complication with 27% of glaucoma, or to Pandey et al. who observed a glaucoma cumulative incidence of 11.7%, we noticed a lower rate of relapse (44%) and complication like glaucoma (5%), which is known to be a severe one [37]. Larger multiethnic studies are needed to explore the severity of the disease according to ethnic origins.

Our study has several limitations. The first is related to the retrospective design of the study, namely missing data; the second limitation is due to non-harmonized diagnoses strategies within the 3 university Hospitals; the third one is due to the small sample size: HLA type was only performed on 10 patients. Complications were also noted for a few patients. Furthermore, we did not notice the necessary time to make a diagnosis after the beginning of symptoms and the necessary time to treat patients. Finally, the treatment was individualized due to the lack of guidelines concerning this disease at time of diagnosis. The high recurrence rate of 44% could be due to sample bias (close follow-up, tertiary centers). Nonetheless, it is the second largest French cohort reported to date [8, 10, 17].

## Conclusion

VKH disease is a rare affection, more present in Asian, Hispanic, and Amerindian populations but in North African populations as well. In French studies patients originated from Maghreb represent a significant proportion, but all ethnicities could and should be involved. Accordingly, we should not limit our vision to patients’ ethnic origins, but diagnosis should be brought up particularly when ophthalmic manifestations are associated to neurological, otologic and/or dermatological signs.

## Supplementary information

**Additional file 1.**

## Data Availability

Not applicable.

## Abbreviations

HLA: Human Leukocyte Antigen
ICGA: Indocyanin Green Angiography
OCT: Optical Coherence Tomography
VKH: Vogt-Koyanagi-Harada
