# Supplementary material for: Vogt-Koyanagi-Harada disease: a retrospective and multicentric study of 41 patients
Source: BMC Ophthalmol. 2020 Oct 7;20:395. doi: 10.1186/s12886-020-01656-x (PMC7539440; doi:10.1186/s12886-020-01656-x)
Supplement: Supplementary file 1 — Additional file 1. [file 12886_2020_1656_MOESM1_ESM.doc]

Supplementary File – Survey

1 / Support center

Name of referring / declaring doctor
E-mail:
Phone:

Fax:
Specialty:

2 / Service in which the patient was primarily followed

Block false answers

3 / Patient identification

a) Sex of the patient:
Man
Woman

b) Ethnicity: specify at 1st or 2nd degree
Japanese
Southeast Asia except Japan
Hispanic
Maghreb
Caucasian
Other

c) Age at diagnosis (in years):

d) First two letters of the name / first two letters of the first name: - - / - -

4 / Monitoring period (between 2000 and 2016, specify the months if possible)

5 / Discovery in phase
Early
Late

6 / Specialty by which the patient entered the care system
Ophthalmology
Internal Medicine
Neurology
Dermatology
ENT
Other (explain, list):

7 / Initial symptom (specify if drop of visual acuity uni or bilateral from the outset)

8 / Signs at the back of the eye
Bilateral retinal serosal detachments
Focal accumulation of subretinal fluid
“Sunset glow fundus”

Sign of Sugiura
Uveitis

9 / What ophthalmological examination(s) did the patient receive?
Fluorescein angiography
Angiography with Indochina green
Optical coherence tomography
Ocular ultrasound
Microscopic ultrasonography
Electro retinogram
Electro oculogram
Visual field
No

10 / Elements found at angiography / OCT
Focal areas of choroidal infusion delay, "pinheads"
Nummular pigmented chorio-retinal scars, "pin points"
Accumulation and / or migration of the retinal pigment epithelium
Diffuse choroidal thickening

11 / Initial visual acuity right eye

12 / Initial visual acuity left eye

13 / Neurological and Auditory Manifestations
Clinical meningeal syndrome
Tinnitus
Hearing loss
Other

14 / Complementary neuro-auditory tests
CSF analysis found pleocytosis
CSF analysis without pleocytosis
Brain scan
Brain magnetic resonance imaging
Audiogram found a decrease of auditory acuity
Normal audiogram
No

15 / Skin manifestations
Alopecia
Poliosis
Vitiligo
Other

16 / Other complementary examinations carried out (and their results)
HIV serology
Syphilis serology
Lyme serology
Research of biological inflammatory syndrome: CRP, fibrinogen
Tuberculosis research: quantiferon / tuberculin IDR / chest X-ray
Connectivity research: ANA, ENA, supplement
ANCA
ACE
Thoracoabdominopelvic CT scan
Biopsy of accessory salivary glands
Other

17 / HLA typing DRB1-04 * 05 / HLA DR4
HLA DRB1-04 * 05 positive
HLA DRB1-04 * 05 negative
HLA DRB1-04 * 05 not sought
HLA DR4 positive
HLA DR4 negative
HLA DR4 not sought

18 / Initial treatment
Bolus of parenteral corticosteroids
Oral corticotherapy
Immunosuppressant
Other
No

19 / Specify the molecule used, the dosage, and the relay

20 / Treatment duration (in months)

21 / Clinical relapse
Yes

No
If yes, how many months after stopping treatment? At what dose of CORTANCYL?

22 / Final eye visual acuity

23 / Final left eye visual acuity

24 / Comments, clarifications

I thank you again for the help provided, hoping that the data are sufficient to achieve a consistent cohort.
